# Social inequalities, regional disparities and health inequity in North African countries

**DOI:** 10.1186/1475-9276-10-23

**Published:** 2011-05-31

**Authors:** Abdesslam Boutayeb, Uwe Helmert

**Affiliations:** 1 Department of Mathematics, Faculty of Sciences, Boulevard Mohamed VI, BP: 717 Oujda, Morocco; 2 URAC04, CNRST-UMP, Boulevard Mohamed VI, BP: 717 Oujda, Morocco; 3 Centre for Social Policy Research, Bremen University, Germany

## Abstract

**Background:**

During the last decades, North African countries have substantially improved economic, social and health conditions of their populations in average. In all countries, human development in general and life expectancy, literacy and per capita income in particular have increased. However, improvement was not equally shared between groups of different milieu, regions or level of income. Social inequalities and health inequity have persisted or even worsened. Data are generally scarce and few studies were devoted to this topic in North Africa as a region. In this paper, we carry out a comparative study on the achievements of these countries, not only in terms of human development and its components but also in terms of inequalities' reduction and health equity.

**Method:**

This study is based on data available for comparison between North African countries. The main data sources are provided by reports released by the World Health Organisation (WHO), United Nations Development Programme (UNDP), United Nations Children's Fund (UNICEF), the World Bank, surveys such as Demographic Health Surveys (DHS) and Multiple Indicator Cluster Surveys (MICS) and finally recent papers published on equity in different countries of the region.

**Results and discussion:**

There is no doubt that education, health and human development in general have improved in North Africa during the last decades. Improvement was, however, uneven and unequally enjoyed by different socioeconomic groups. Indeed, each country included in this study shows large urban-rural disparities, discrepancies between advantaged and disadvantaged regions and cities; and unacceptable differences between rich and poor. Health inequity is particularly seen through access to health services and infant mortality.

**Conclusion:**

During the last decades, North African decision makers have endeavoured to improve social and economic conditions of their populations. Globally, health, education and living standard in general have substantially improved in average. However, North African countries have still a long way to go to reduce social inequalities and health inequity at different levels: rural-urban, advantaged-marginalised regions and cities, between groups of different level of income and wealth. The challenge for the next decade is not only to improve economic, social and health conditions in average but also and mainly to reduce avoidable inequalities in parallel.

## 1. Introduction

During the last decades, North African countries have seen a substantial improvement in the living standard of their populations. Life expectancy, literacy and per capita income improved in all countries and consequently, human development index has been steadily increasing. Beyond the global trend, however, improvement was not equally enjoyed. Indeed, urban-rural disparities, discrepancies between regions and inequalities among socioeconomic groups have persisted or even increased in North African countries during the last decades.

North African countries, namely, Algeria, Egypt, Libya, Morocco and Tunisia are often included in other regions like the Arab World, Africa, World Health Eastern Mediterranean Region (WHO-EMRO) and Middle East and North Africa (MENA). Belonging at the same time to Africa, Mediterranean Region, Arab World and Islamic countries, North African countries share language, religion and social and cultural custom. Globally, these countries are engaged in a multidimensional transition (demographic, economic, epidemiological and geographic) (Table 1) [1]. To our knowledge, in each of these countries, few published studies were related to this topic [2-5] and fewer papers have dealt with the region as a whole [6,7]. In this paper, we carry out a comparative study on their achievements not only in terms of human development but also in terms of inequalities reduction and health equity.

**Table 1:** North African countries: Demographic data as published by UNDP in 2010

	Algeria	Egypt	Libya	Morocco	Tunisia
Population (in million)	35.4	84.5	6.5	32.4	10.4
% of Urban population	66.5	43.4	77.9	58.2	67.3
GDP per capita (PPP$)	8320	5889	17068	4628	7979
Adult literacy (%)	72.6	66.4	88.4	56.4	78
Median age	26.2	23.9	26.2	26.2	29.1
Fertility (births per woman)	2.3	2.7	2.5	2.3	2.8
Life expectancy (years)	71.7	70.7	73.4	70.4	73.5
Infant Mortality Ratio (IMR) (Deaths per 1000 live births)	36	20	15	32	18
Maternal Mortality Ratio (MMR) (Deaths per 100,000 live births) 180	108	130	97	240	100

## 2. Human development: similarities and disparities

The introduction by the United Nations of human development index (HDI) as a mean of three indicators weighed equally: health (life expectancy at birth), standard of living (purchasing power parity income) and education (literacy and enrolment) illustrated clearly the importance of health and education and proved that an increase in national income alone does not capture development in its fullest sense [8,9]. As part of the Arab region, North African countries were concerned by the five Arab Human Development Reports [10-14]. While noting the substantial economic and social progress made during the last decades, the previous reports stressed that North African countries and Arab countries in general have accomplished less than expected in terms of human development globally and in education, health and social justice in particular.

In a previous paper dedicated to human development and health indicators in the Arab region [5], the authors carried out data analysis on education and health measures (omitting income), focusing on the link between human development and health indicators. Compared to oil-rich countries, North African countries were seen to have low rates of literacy and unacceptable low rates of access to health care and facilities, especially in rural areas.

**Figure 1:**
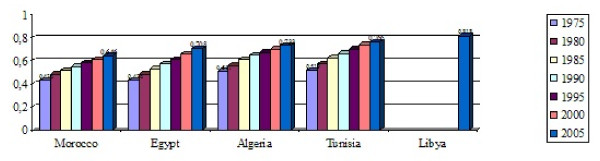
**Evolution of human development index in North African countries**. All North African countries had a similar increasing trend in human development index

Analysing the evolution of human development in North Africa, it appears that, during the last three decades, all countries had a similar increasing trend in human development index, although data is partly missing for Libya (Figure 1).

From 1980 to 2010, Morocco achieved the best relative increase in human development index (61%) whereas Algeria had the lowest relative increase (53%). Egypt and Tunisia accomplished nearly the same relative increase (58% and 57% respectively). According to the Human Development Report 2010 [1], Libya (53^d^), Tunisia (81^st^) and Algeria (84^th^) belong to the High Human Development group whereas Egypt (101^st^) and Morocco (114^th^) fall in the medium group. It should be noted, however, that UNDP has made some changes in the way of computing HDI (shifting from arithmetic mean to geometric mean) and also in the classes by HDI (Very high group, high group, medium group and low group).

The human development index being an average of three components, more details can be obtained by looking at the achievement of each country separately on health, education and income. In 2010, all North African countries had a mean life expectancy at birth greater than 70 years (Table 1). If we consider, however, the expectation of lost healthy years, these numbers will be amputated by 10 years or more.

In terms of literacy and education achievements, North African countries embarked upon the third millennium burdened by millions of illiterate adults. For instance, in 2010 Morocco had nearly half of the adult population illiterate and around 60% of combined gross enrolment in primary, secondary and tertiary education. For Egypt, Algeria, and Tunisia adult literacy rates were respectively 66.4, 72.6 and 78 whereas Libya had a rate of 88.4%. Similarly, the rates of combined gross enrolment in primary, secondary and tertiary education where around 75% for Algeria, Egypt and Tunisia, and a higher rate for Libya (94.1%) [1,14].

Looking at the between countries difference in income, it appears that GDP per capita in Tunisia ($7979) is much higher than in Morocco ($4628) and Egypt ($5889) and, although Algeria and Libya are both oil-countries producers, the Algerian GDP per capita ($8320) is less than half that of Libya ($17068).

As a conclusion, human development in North Africa is relatively uneven. For instance, life expectancy in Libya and Tunisia is three years higher than life expectancy in Egypt and Morocco. In terms of adult literacy, the gap between Libya and Morocco is 32% and finally the Libyan GDP per capita is 3 times higher than GDP in Egypt and 3.7 times higher than GDP in Morocco.

## 3. Inequalities and inequity within countries

Human development index is among the most used indicator giving a summary measure of Human development and allowing for comparison between countries around the world. However, although HDI deals with achievements in education, health and income in a given country, measurements are national average numbers which may hide inter-groups inequalities and regional disparities. Different forms of inequity remain not captured even with the use of measurements such as human poverty index (HPI), gender-related development index (GDI) and gender empowerment measure (GEM).

### 3.1 Measures of inequality

Consumption or income index is often used to measure economic inter-groups inequalities. For pragmatic reasons, however, wealth index is becoming the most used in research on economic disparities. This tendency is justified by the use of data collected through demographic health surveys (DHS) which contain no information on consumption and income but on the other hand, they do have sufficient data on assets necessary for a decent living standard and wellbeing (housing, access to water and sanitation, health services and health outcomes, education, employment, violence, leisure, etc...). Nevertheless, neither consumption/income nor wealth index is sufficient to define the multidimensional inter-group inequalities. Disparity and inequity can also be measured through education, gender, place of residence and other factors like ethnicity and stigma [15].

### 3.2 Inequality in income or consumption in North Africa

**Figure 2:**
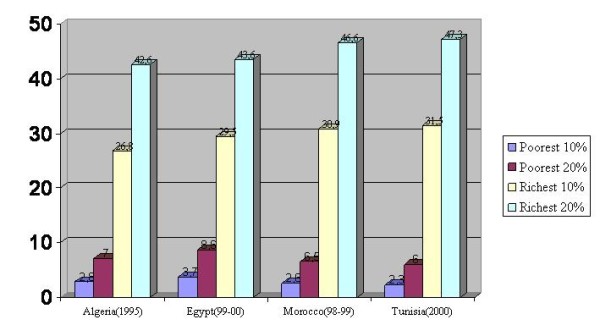
**Inequality in the share of income or consumption **[14]. Sharp inequality in the share of income/consumption

North African countries have a similar pattern of income/consumption inequality. According to the last Arab Human Development Report [14], in Morocco and Tunisia, the richest 20% absorb nearly 50% of all income/consumption, compared to 6.5% or less for the poorest 20%. The ratio of richest 10% to poorest 10% reaches 13.4 in Tunisia (Morocco: 11.7, Algeria: 9.6, Egypt: 8) (Figure 2). It is worth stressing that Tunisia which was seen to have better human development index than Algeria, Egypt and Morocco, is revealed as the country with the worst share of income/consumption. Globally, the four countries have unacceptable inequality in the share of income/consumption. This uneven distribution will certainly have consequences on the rates of poverty and access to basic social services like education and health but also on psycho-socio behaviours in general (violence, marginalisation, mental illness, etc..). No Development can be sustainable with such unfair inequalities.

### 3.3 Inequality measured by Gini Index

Gini index has been widely used, especially by economists, to measure inequalities between different socioeconomic groups. The value of this index is between 0 and 1 (large values indicate large level of inequality). As shown in Table 2, Arab countries in general and North African countries in particular, have a high Gini index compared to other developed and developing countries. Moreover, in almost all North African countries, this index has increased during the last decade or so.

**Table 2:** Evolution of Gini index in some Arab countries

Algeria	35.3	(1995)		
Egypt	30.1	(1995)	32.1	(2004)
Morocco	39.5	(1998)	40.9	(2007)
Tunisia	41.7	(1995)	37.7	(2005)
Mauritania	37.3	(1995)	39.0	(2000)
Jordan	36.4	(1997)	37.7	(2006)
Syria	33.7	(1997)	37.4	(2004)
Yemen	33.4	(1998)	37.7	(2005)
Ethiopia	30	(2000)		
Finland	26.9	(2000)	26.8	(2008)
Romania	31.0	(2000)	32.0	(2008)
South Korea	31.6	(1998)	31.4	(2009)
Sweden	25.0	(2000)	23.0	(2005)
European Union			31.0	(2006)

### 3.4 Inequality in education and literacy

#### 3.4.1 Education

Since the launch of the Millennium Development Goals [16], North African countries made noticeable achievements in terms of primary education enrolment for both boys and girls. Considering, however, the combined enrolment ratio for primary, secondary and tertiary education shows that the rates are around 75 for Algeria, Egypt and Tunisia, with a small difference between male and female whereas for Morocco the rates are 55% for females and 62% for males. These figures stress that efforts made to increase primary enrolment may be hampered by high rates of dropping out of school, child labour and difficult access to secondary and tertiary education especially for poor girls and/or those living in rural areas. Indeed, in Egypt, a global study on child poverty and disparities recently conducted by UNICEF showed that 1/5 of children live in poverty and that one in four children are deprived of one or more dimensions of welfare. Stressing that vulnerability is the same for boys and girls, the study indicates that child-poverty in Egypt is regional, with higher concentrations in rural areas and Upper Egypt. For instance more than 30% of children in rural areas live in households that are poor compared with 12.6% in urban areas [17]. The consequences for education are obvious since a child born in the poorest quintile is 11 times likely to be deprived from education than a child born in the richest quintile.

In Morocco, the educative system efficiency is very low (57.5 for primary school and 35.4 for secondary level). The lost is due to high levels of dropping out and repetition. Analysis of data in the poorest 404 rural districts identified by the National Initiative for Human Development (NIDH) revealed that socioeconomic conditions and proximity of school are the main factors explaining dropping out [18].

In Tunisia, a multivariate analysis of data from MICS3 showed that the proportion of children reaching the fifth level of primary school was principally correlated with milieu and children work [19].

#### 3.4.2 Literacy

Despite the efforts devoted by North African countries during half a century in order to reduce the rate of illiteracy, the problem is still burdening these countries with more or less acuteness as illustrated by the following examples.

##### Example 1. Literacy rate in Morocco (1999)

**Figure 3:**
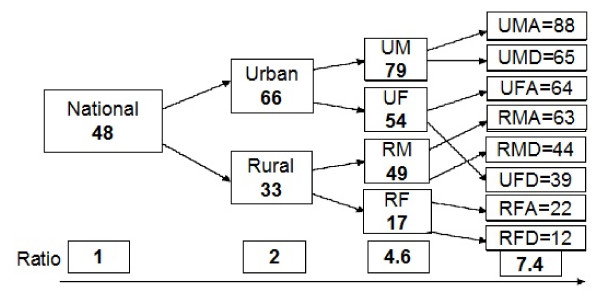
**Literacy in Morocco**. A rural poor woman cumulates three "handicaps" by gender, milieu and income

As shown by the diagram below (Figure 3), there may be an overlapping and cumulative effect of inequities. With a national rate of 48**%**, the urban literacy rate is twice that of rural areas, then urban males are 4.6 likely to be literate than rural females, and at a third level, advantaged (rich) urban males are 7.4 likely to be literate than disadvantaged (poor) rural females. Consequently, a rural poor woman cumulates three "handicaps" by gender, milieu and income.

##### Example 2. Evolution of illiteracy in Algeria

**Figure 4:**
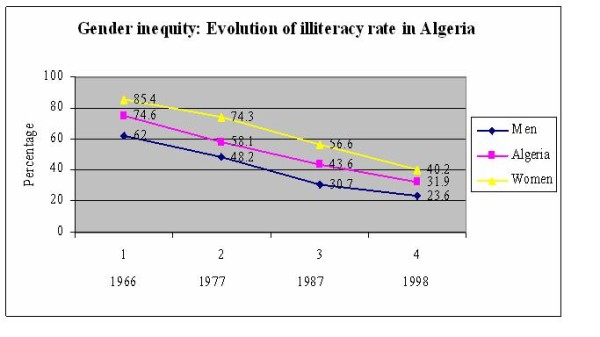
**Evolution of illiteracy rates in Algeria **[18]. Absolute and relative gender inequality in education

When dealing with inequalities, one must be careful about the tool used to measure such inequalities (absolute, relative, index of inequality, whole gradient, etc...). Indeed, the same dataset may lead to opposite conclusions quantitatively and qualitatively as illustrated by the following diagram on the evolution of men and women illiteracy in Algeria from 1966 to 1998 (Figure 4) [19].

This diagram shows that, although illiteracy decreased steadily for both sexes, women remained more illiterate than men during three decades. Moreover it is not easy to say if the gender gap was reduced or not. In fact this dataset may lead to opposite conclusions. For instance, the calculation shows that the absolute gap was reduced but relatively, inequity increased between men and women (Table 3).

**Table 3:** Absolute or relative differences: which to choose?

	1966	1998	Trend
Women	85.4	40.2	

Men	62.0	23.6	

Absolute differences	**23.4**	**16.6**	**Decreasing**

Relative differences	**1.37**	**1.7**	**Increasing**

## 4. Health inequity

### 4.1 General pattern

**Figure 5:**
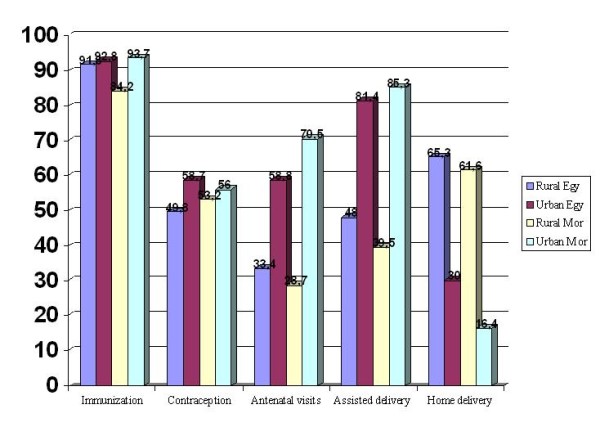
**Rural-urban access to health services in Egypt and Morocco around 2005 **[21,22]. Egypt and Morocco: inequity in antenatal visits, assisted births by skilled personnel and births given at home.

Data from Demographic Health Surveys in North African countries indicate that for health services like immunization coverage and contraception, inequalities are generally attenuated between rural and urban areas, rich and poor families, as well as between developed and deprived regions. This achievement is mainly due to national and international efforts based on generalised policies of public health with specified targets aiming to reach deprived and vulnerable populations. At the opposite side, other health services like antenatal visits, assisted births by skilled personnel and births given in health centres, as well as health outputs like infant and child mortality, stunting and underweight all show unjustifiable gaps between rural and urban; poor and rich; developed and deprived regions; and illiterate and educated women. An illustration is given by Figure 5 for rural-urban access to health services in Egypt and Morocco [20,21].

### 4.2 Rural-urban and/or regional inequalities

In general, all North African countries show rural-urban and/or regional discrepancies in health indicators and access to care.

According to a review on social determinants of health carried out by WHO in seven countries in the Eastern Mediterranean Region, health indicators vary regionally between Lower Egypt and Upper Egypt, and, in each region, discrepancies are found between rural and urban populations [22]. Similarly, in Morocco, more than 30% of the rural population has to travel at least 10 kilometres to reach the nearest health facility, the number of inhabitants per physician ranges from 6362 in the rural area of Taounate (in the remote north east) to 380 in the capital, Rabat. The number of public hospital beds per 100 000 population ranges from 31 in the rural area of Berkane (in the remote north-east), to 444 in Rabat [2,22]. As shown in Figure 5, A Moroccan rural woman is twice unlikely to attend antenatal care or to deliver with assistance of medical personnel; and nearly four times likely to deliver at home than a Moroccan urban woman. For Egyptian women, the ratios are respectively 0.5, 0.6 and 2.2. In Libya, effect of socioeconomic factors on child development were considered in a cross sectional study carried out in two regions (Al Jabel and Tripoli) of the Jamahiriya on the growth and nutritional status of children under five years of age. The prevalence of stunting was higher among Al Jabel children (6.1%) than in Tripoli (2.5%) and in rural (6.8%) rather than in urban areas (2.8%) [5].

**Figure 6:**
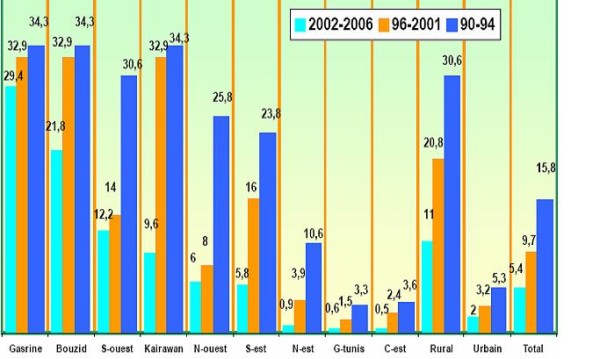
**Non assisted deliveries in Tunisia**. Health inequity has increased during the last decade between governorates

According to data provided in Tunisia by four surveys spanning more than a decade period (PAPCHILD 1994, PAPFAM 2001, MICS2-2002 and MICS3-2006) [19,23], health indicators related to women and children have improved substantially in global terms. However, large gaps remain essentially according to wealth's group and milieu of residence (rural-urban and between governorates). This result was particularly confirmed by a logistic regression model in MICS2, showing that antenatal care, postnatal care, births assisted and in health facilities, vaccination, child malnutrition all exhibit significant correlation with milieu and mothers education level. Illustration is given in Figure 6 for the percentage of deliveries without assistance in different governorates.

This figure shows clearly the gap between different governorates for all three surveys. Moreover, health inequity has increased during the last decade as indicated in Table 4 for the percentages of deliveries without assistance in different regions. A straightforward analysis of data shows that, although at national level the number has been steadily decreasing between 1994 and 2006, the decrease was uneven between urban and rural and also between cities and regions.

**Table 4:** Evolution of the rate of non assisted deliveries in Tunisia

	1994	2001	2006
Rural	30.6	20.8	11

Urban	5.3	3.2	2

Ratio R/U	5.8	6.5	5.5

Gasrine	34.3	32.5	29.4

G-Tunis	3.3	1.5	0.6

Ratio	10.5	22	49

National	15.8	9.7	5.4

National trend		Decrease by 1.6	Decrease by 1.8

### 4.3 Poor-Rich inequalities

Few data on rich-poor health inequity in North Africa are published. However existing evidence indicates that exacerbated inequalities are found in the use of health services such as antenatal visits, births assisted by skilled medical personnel and births given in a medical centre. Consequently, indicators like infant mortality and maternal mortality will show gaps between rich and poor.

In Morocco, according to data from Demographic Health Surveys (DHS 2003-2004) and summarised in Table 5, a poor woman is 7 times unlikely to receive antenatal care than a rich woman, the ratios for giving birth at home and delivery with assistance of a traditional midwife are respectively 11.75 and 12.9. For postnatal visits the ratio is 1.3 but it should be stressed that among all women, around 90% had no postnatal visit [20].

**Table 5:** Access to health care facilities and health personnel in Egypt and Morocco [20,21]

Socioeconomic categories	Women who did not receive antenatal care		Women who had no postnatal visit		Women who gave birth at home		Women who delivered with assistance of a traditional midwife	
	Morocco	Egypt	Morocco	Egypt	Morocco	Egypt	Morocco	Egypt
	2003/04	2008	2003/04	2008	2003/04	2008	2003/04	2008
Urban	15.1%	15.0%	83.7%	18.6%	16.4%	14.5%	7.7%	9.1%
Rural	52.1%	33.1%	96.4%	41.5%	61.1%	36.3%	33.8%	25.9%
Ratio R/U	3.5	2.2	1.2	2.2	3.7	2.5	4.4	2.8

Poorest quintile	60.3%	46.5%	97.1%	47.5%	70.5%	54.6%	39.9%	41.4%
Middle quintile	29.4%	26.3%	89.9%	32.8%	31.8%	25.9%	14.1%	16.5%
Richest quintile	6.9%	7.6%	73.6%	9.4%	6.0%	5.4%	3.1%	2.7%
Poorest/Richest	8.7	6.1	1.3	5	11.75	10	12.9	15

In Egypt, more recent data from DHS 2008 indicate that the gaps between rich and poor women who did not receive antenatal care, had no postnatal visit, gave birth at home or delivered with assistance of a traditional midwife are similar to those in Morocco. The ratios are respectively 6.1, 5, 10 and 15 (Table 5) [21].

**Figure 7:**
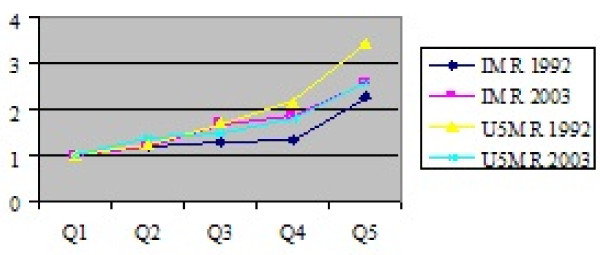
**Health outputs in Morocco **[21]. The poorest children (respectively infants) are three time likely to die than the richest children and infants

The same data source (DHS) shows also that, for under five mortality rates, poorest children (respectively infant) are three times likely to die than the richest children and infant. Moreover, health inequity is increasing or at least persistent as indicated by Figure 7 for infant mortality in Morocco [20]

In Egypt, the proportion of the richest women having multiple antenatal visits and that of women giving birth in presence of skilled personnel are threefold that of the poorest women. The gap in births given at home is fivefold. The poorest children (respectively infant) are two and half time likely to die than the richest children and infant. Stunting and under weight reveal similar levels of inequality [21]. More generally, it is estimated that 7 million children are deprived of one or more of their rights, which include nutrition, access to basic health care, education and shelter [17].

Analyzing Egyptian data provided over a ten-year period by the Demographic Health Surveys (EDHS1995, EDHS2000 and EDHS2005), Khadr has shown that, although, almost all maternal health indicators have globally improved, improvements were not equally enjoyed by all population groups [4]. For instance, considering the percentages of prenatal care, skilled birth attendant and delivery at home, the study showed persistence of inequities among women of different levels of wealth and education as indicated by the concentration index (CI) in Table 6.

**Table 6:** Disparities in delivery, Egypt (1995-2005) [4]

	Year		
	
Indicator	1995	2000	2005
Any prenatal care	42.40	54.10	71.40
CI by Education	0.41	0.39	0.41
CI by Wealth index	0.50	0.37	0.45
Skilled birth attendant	41.70	55.80	70.50
CI by Education	0.41	0.38	0.37
CI by Wealth index	0.55	0.42	0.47
Delivery at home	64.50	49.10	33.60
CI by Education	-0.42	-0.37	-0.35
CI by Wealth index	-0.53	-0.41	-0.47

(CI: Concentration Index)

## 5. Conclusion

During the last decades, North African countries have seen a noticeable growth in terms of economic, social and health indicators [2-4,24]. Unfortunately, this growth has not been enjoyed equally by different socioeconomic groups of the same country. Sharp social inequalities and health inequities are found between rural and urban, regions and wealth income groups. For instance, social status is a major determining factor of survival for children. In North African countries, illustration is particularly given by post-natal mortality which is mainly due to factors such as food, primary health care and hygiene. It is striking to see that in these countries, postnatal mortality may be five times greater in children belonging to the poorest quintile, compared to children living in the richest quintile. Similarly, a child of an illiterate woman is three times more likely to die than a child of a woman with secondary or higher level of education, and finally, post natal mortality is 2.5 times greater in rural areas than in urban cities.

As stressed by the WHO Commission on Social Determinants of Health in its report entitled "closing the gap", where systematic differences in health are judged to be avoidable by reasonable action, they are quite simply unfair [25]. Like other developing countries, North African countries are struggling to reach the Millennium Development Goals by 2015. But in doing so, they may improve average indicators with persistent or even increasing inequalities. Moreover, facing the double burden of communicable and non communicable diseases with limited health budget, North African decision makers need to adopt optimal and efficient strategies. Health decisions need to focus on targeted and equitable health programmes that aim to enhance the mean status of the whole population but at the same time to reduce regional disparities between developed and disadvantaged regions; inequalities between rich and poor, and marginalisation of the rural population. In the light of the general uprising affecting most of Arab and North African countries, decision makers are urged to act on unjustifiable and avoidable inequalities, otherwise they will have no (or very few) chances to achieve a sustainable development.

## Competing interests

The authors declare that they have no competing interests.

## Authors' contributions

Having contributed equally to this work, the two authors also read and approved the final

manuscript
